# Assessing Changes in Bacterial Load and Antibiotic Resistance in the Legon Sewage Treatment Plant between 2018 and 2023 in Accra, Ghana

**DOI:** 10.3390/tropicalmed8090427

**Published:** 2023-08-28

**Authors:** Raymond Lovelace Adjei, Lady Asantewah Boamah Adomako, Appiah Korang-Labi, Franklin Kodzo Avornyo, Collins Timire, Rita Ohene Larbi, Cletus Kubasari, Stephen E. D. Ackon, Anthony Reid

**Affiliations:** 1 Council for Scientific and Industrial Research—Animal Research Institute, Accra P.O. Box AH 20, Ghana; favornyo@yahoo.com (F.K.A.); rolarbi@csir.org.gh (R.O.L.); 2 Council for Scientific and Industrial Research—Water Research Institute, Accra P.O. Box AH 38, Ghana; asantewa84@gmail.com; 3 Department of Medical Microbiology, University of Ghana Medical School, Accra P.O. Box GP 4236, Ghana; guylabi2@gmail.com; 4 International Union against Tuberculosis and Lung Disease (The Union), 2 Rue Jean Lantier, 75001 Paris, France; collins.timire@theunion.org; 5 Research and Development Division, Kintampo Health Research Centre, Ghana Health Service, Kintampo P.O. Box 200, Ghana; kubasari.cletus@kintampo-hrc.org; 6 Accra Sewerage Improvement Project, Accra Metro Sewerage Unit, Ministries, Accra P.O. Box MB 201, Ghana; 7 Operational Research Unit (LuxOR), Medical Department, Médecins Sans Frontières Operational Centre, 1050 Bruxelles, Belgium; tony.reid@brussels.msf.org

**Keywords:** antimicrobial resistance (AMR), sewage treatment plant (STP), *E. coli*, effluent, influent

## Abstract

Wastewater treatment plants are efficient in reducing bacterial loads but are also considered potential drivers of environmental antimicrobial resistance (AMR). In this study, we determined the effect of increased influent wastewater volume (from 40% to 66%) in the Legon sewage treatment plant (STP) on the removal of *E. coli* from sewage, along with changes in AMR profiles. This before and after study compared *E. coli* loads and AMR patterns in influent and effluent samples from a published baseline study (January–June 2018) with a follow-up study (March–May 2023). Extended-spectrum beta-lactamase (ESBL) *E. coli* were measured pre- and post-sewage treatment during the follow-up study. The follow-up study showed 7.4% and 24% ESBL *E. coli* proportions in influent and effluent, respectively. In both studies, the STP was 99% efficient in reducing *E. coli* loads in effluents, with no significant difference (*p* = 0.42) between the two periods. More *E. coli* resistance to antimicrobials was seen in effluents in the follow-up study versus the baseline study. The increased influent capacity did not reduce the efficiency of the STP in removing *E. coli* from influent wastewater but was associated with increased AMR patterns in effluent water. Further studies are required to determine whether these changes have significant effects on human health.

## 1. Introduction

Globally, increasing antimicrobial resistance (AMR) is a public health threat to humans, animals, and the environment. The environmental aspect of AMR, until recently, had been given less attention [1,2], but the World Health Organization (WHO) recommends a One Health approach for addressing increasing AMR [3]. This is a multidisciplinary approach that requires the combined efforts of stakeholders in human, animal, and environmental health [1]. Evidence of increasing AMR in the environment has recently sparked interest in chemical, physical, and biological ways to properly dispose of human waste, which in turn aims to improve the effects of human waste on the environment and reduce AMR [4].

Managing human waste relies on sewage treatment plants (STPs) as the most important component to maintain water safety in the environment for humans, animals, and agriculture. Effluent from urban STPs eventually finds its way into existing water bodies [5]. Properly maintained and functioning STPs are crucial to reducing environmental pollution.

However, STPs are considered potential reservoirs for AMR development and spread because they concentrate on human and animal pathogens [6,7,8]. The ponds within the treatment plants allow enough time for the exchange and transfer of resistant genes among the bacteria present in the pond [9,10].

In Ghana, sanitation improvement has been a major challenge [11] mostly due to lack of financial resources. Efforts by the government, with the help of donor partners, to improve this situation have resulted in the building of 11 STPs [12]. Unfortunately, only three were fully operational at the time of the study [13].

In 2012, the Legon STP was built as part of the Accra sewage improvement project with the aim of providing an improved sewage and sanitation system for disposing of wastewater in Accra [12]. The effluents from the plant are discharged into the Onyasia stream [14], which serves as a source of irrigation for some vegetable farms, emphasising the importance of good performance.

A baseline study (January to June 2018) carried out on the Legon STP [14] focused on the *E. coli*, *Pseudomonas aeruginosa*, and *Aeromonas hydrophila* loads pre-and-post-treatment, as well as the resistance profiles of commonly used antibiotics. In an era of heightened concern over AMR, this was an important step in identifying the spectrum of resistance among bacteria being discharged into the nearby stream [14].

At the time of the baseline study (January–June 2018), the treatment plant was operating at 40% capacity (volume) but still managed to remove 99% of bacteria from the influent. However, the bacterial load in the effluent still did not meet Ghana standards for wastewater discharge (*E. coli* at 10 CFU/mL and 400 CFU/mL for total coliforms). The baseline study also found significant resistance to some commonly used antibiotics.

Following the publication of the baseline study, the results were presented to stakeholders in the Environment and Wastewater Management and the AMR Committees of Ghana. Recommendations were made that included increasing the intake volume of the Legon STP, which could further improve its efficiency.

Currently, the capacity (volume) of the Legon STP has been scaled up to about 66% through trucked-in waste, and this increased capacity was expected to improve the clearance of *E. coli* and other bacteria. This is because wastewater stabilisation pond performance can be influenced by hydraulic performance, short-circuiting, sludge accumulation, and flow velocities [15]. We believed that it would, therefore, be useful to compare the efficiency of the treatment plant five years later to determine whether the overall bacterial load in the effluent was reduced further and to see how antibiotic resistance patterns had evolved since 2018. We expected that the increase in wastewater intake (volume) of the plant would result in greater bacterial removal efficiency.

To assess AMR, *E. coli* and extended-spectrum beta-lactamase (ESBL) *E coli* levels were determined in the follow-up study. *E coli* was chosen as a marker because it is an indicator of wastewater quality, analysis is relatively inexpensive, simple to apply, and methodologies are accessible. *E. coli* and ESBL *E. coli* can serve as markers for monitoring antimicrobial resistance in wastewater [16].

The aim of the follow-up study was to describe changes in the *E. coli* loads and AMR profiles of the influent and effluent wastewater before and after an increase in intake capacity (volume) of the Legon STP, Accra, Ghana, between January to June 2018 (baseline) and, March to May 2023 (follow-up). The findings from this follow-up study could be important to convince authorities to further increase the sewage intake (volume) of the Legon STP and to seek funds to reactivate other dormant STPs in Accra.

## 2. Materials and Methods

### 2.1. Study Design

This was a before-and-after, cross-sectional study that compared laboratory data from wastewater samples obtained from the Legon STP in Accra, Ghana, from January 2018 to June 2018 (baseline) and March 2023–May 2023 (follow-up).

### 2.2. Study Setting

Accra, the capital of Ghana, now has three municipal STPs in operation: Legon, Mudor, and Adjen Kotoku. Currently, less than a quarter of the STPs are fully operational [14]. Built in 2012, the Legon STP has the capacity to process 9000 m^3^ of sewage daily [17]. In 2018, the Legon STP received about 3600 m^3^ but currently receives 6000 m^3^ of wastewater daily [17], an increase from 40% to 66% efficiency. The treatment plant is made up of a succession of open waste stabilisation ponds: comprising three anaerobic, three facultative, and six maturation ponds. The plant receives wastewater from Achimota Hospital, Achimota Senior High School, University of Ghana, Achimota Basic School, University of Professional Studies, and the Presbyterian Senior High School. The effluent from the treatment plant is directly discharged into the Onyasia stream [5].

### 2.3. Baseline Study, Dissemination, Recommendations and Follow-Up

Following completion of the baseline study (January–June 2018), the following activities took place: 1. publication of the findings in an open-access journal (Tropical Medicine and Infectious Disease); 2. presentation of findings to the Ghana AMR committee, including health, animal, and environmental sector representatives; and 3. addition of the Head of Liquid Waste Management, Accra Metropolitan Assembly as a co-author. Several recommendations were made in the paper, including 1. educating farmers on the nature of the Onyasia River as a potential threat to irrigation; 2. assisting farmers on the use of protective clothing; 3. increasing plant operating capacity (volume); and 4. reactivating non-functioning treatment plants. Although most of these recommendations were not acted upon, the capacity of the Legon STP was increased independently of the baseline study recommendations.

### 2.4. Study Population and Period

Both the baseline and follow-up studies collected 12 wastewater samples (six each for influent and effluent) from January to June 2018 (baseline) and March 2023 to April 2023 (follow-up) at the Legon STP.

### 2.5. Sample Collection and Laboratory Analysis

Wastewater sample collection and analyses were performed according to the Standard Methods for Examination of Water and Wastewater [18] from March to May 2023. In this study, the wastewater samples were collected every two weeks (a total of 12 samples) into sterile 500 mL bottles from two sampling points as follows: 1. influent (untreated sewage) entering the treatment plant, 2. effluent (treated sewage), exiting the plant. All samples were collected in duplicate and were immediately placed on ice and transported to the Council for Scientific and Industrial Research—Water Research Institute (CSIR-WRI) microbiology laboratory. The samples were analysed within two hours of arrival at the laboratory.

Following a ten-fold serial dilution in phosphate-buffered saline solution, all wastewater samples were analysed using membrane filtration on tryptone bile X-glucuronide (TBX) agar (Oxoid) for *E. coli* and TBX supplemented with 4 µg/mL cefotaxime (TBX/CTX) for ESBL *E. coli.* Inoculated plates were incubated at 37 °C for 18–24 h. Plates with counts between 30–300 blue-green colonies cultured from both TBX (Oxoid) and TBX/CTX were counted and recorded as colony-forming units per 100 mL (CFU/100 mL).

*E. coli* isolates were further confirmed with the indole test. Five presumptive ESBL *E. coli* which grew on TBX/CTX plates were streaked for purity on TBX agar and then on Nutrient Agar (Himedia) and confirmed using the double-disc diffusion method according to Clinical and Laboratory Standards Institute (CLSI, 2019) using Cefotaxime 30 µg, Ceftazidime 30 µg, and Ceftazidime 30 μg/Clavulanic Acid 10 µg Cefotaxime 30 μg/Clavulanic Acid 10 µg (Becton Dickenson, Franklin Lakes, NJ, USA) [19]. ESBL *E. coli* over *E. coli* ratio was estimated using ISO 8199 Water Quality-General guidance on the enumeration of micro-organisms by culture [18]. The percentages of ESBL were calculated according to the formula

$$\mathrm{ESBL}\%=\frac{\left(\mathrm{ESBL}\;\mathrm{count}\right)}{\mathrm{Total}\;E.\;\mathrm{coli}}\;\times \;100$$

In the baseline study, a total of sixty isolates were used for antimicrobial susceptibility testing. To enable easy comparison with the baseline study, the follow-up study also used a total of sixty confirmed *E. coli* isolates that were subjected to antimicrobial susceptibility testing by the Kirby Bauer disc diffusion method according to the CLSI, 2019 guidelines [19]. Antibiotics tested included those recommended in the CLSI 2019 guidelines. These included ciprofloxacin 5 µg, tetracycline 30 µg, amoxicillin/clavulanate 20/10 µg, cefuroxime 30 µg, aztreonam 15 µg, meropenem 10 µg and gentamicin 10 µg (Becton Dickenson, Franklin Lakes, NJ, USA). After 18–24 h, zones of inhibition were measured, and the isolates were categorised as resistant and sensitive in accordance with the CLSI guidelines [19].

### 2.6. Quality Control Procedures

All bacteriological media were made in accordance with the manufacturer’s requirements. Before usage, each batch was tested for sterility and the potential to support *E. coli* growth. Sterile distilled water was used as a negative control, whilst *E. coli* American type culture collection (ATCC 25922) and *Klebsiella pneumoniae* national collection of type culture (NCTC) 133668 were used to ensure the quality of the antimicrobial susceptibility assay [19].

### 2.7. Data Variables, Sources of Data, and Validation

Data from baseline study were imported for analysis and comparison with the follow-up study. Data variables in the follow-up study were sample sources (influent and effluent), *E. coli* counts, *E. coli* resistance profiles, and proportions of ESBL *E. coli.* Data on sample collection points and sources, *E. coli* loads, and resistant profiles were entered into a laboratory notebook and then double-entered into a database (Microsoft Excel^®^ v. 2307) that was kept in the laboratory. Data from the baseline study was already available.

### 2.8. Statistical Analysis

Bacterial loads and resistance patterns were reported using descriptive statistics with Microsoft Excel v. 2307 (Redmond, WA, USA, Microsoft Corporation 2013). The thresholds, 10 CFU/100 mL for *E. coli* and 400 CFU/100 mL for total coliforms, are the acceptable limits set by the Ghana Environmental Protection Agency (EPA) for effluent discharge. Data were analysed using Stata version 13.0 (StataCorp, College Station, TX, USA). Categorical data were summarised using frequencies and proportions. Initially, continuous data were assessed for normality using the Shapiro–Wilk test. As data were skewed, median CFU/100 mL and interquartile ranges (IQRs) were presented. Median CFU/100 mL for influent were compared between the baseline and follow-up periods using the Wilcoxon rank sum (Mann–Whitney U) test. The significance level was set at *p* ≤ 0.05.

## 3. Results

### 3.1. E. coli in Wastewater Samples from the Legon STP, Accra, during the Baseline and Follow up Studies

Table 1 shows a comparison of the total *E. coli* loads in wastewater samples between January to June 2018 (baseline) and March to May 2023 (follow-up) studies. In both studies, there was a large reduction (99%) in *E. coli* load in effluent compared to influent wastewater.

### 3.2. Antibiotic Resistance in E. coli from Influent and Effluent Wastewater Samples

Sixty *E. coli* isolates (30 each) from influent and effluent wastewater samples were screened against the seven antibiotics. Meropenem was used in place of imipenem in the follow-up study.

**Figure 1:**
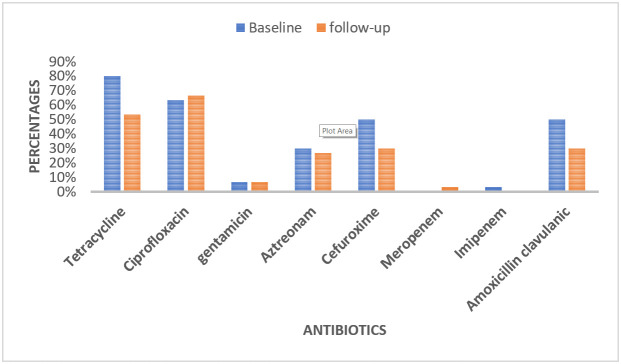
Proportions of resistant *E. coli* in influent wastewater samples from the Legon sewage treatment plant, Accra, baseline (January–June 2018) and follow-up (March–May 2023).

Figure 1 shows the proportion of antibiotic resistance of *E. coli* from influent wastewater samples during the baseline and follow-up studies. There was less resistance to four antibiotics (tetracycline, amoxicillin–clavulanic, aztreonam, and cefuroxime) in the follow-up study compared to the baseline, slightly more resistance to ciproflaxacin but no change in observed gentamicin and meropenem resistance.

**Figure 2:**
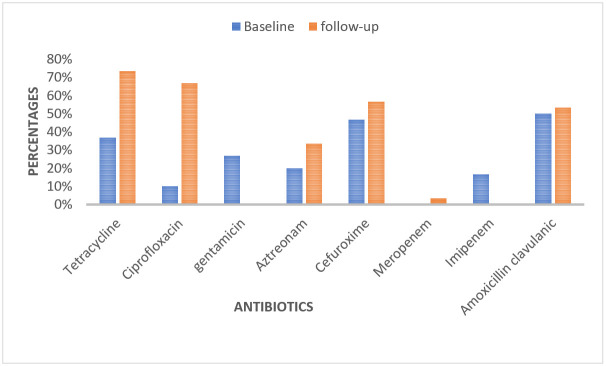
Proportions of resistant *E. coli* in effluent wastewater samples from the Legon sewage treatment plant, Accra, baseline (January–June 2018) and follow-up (March–May 2023) studies.

Figure 2 shows the proportion of antibiotic resistance of *E. coli* from effluent wastewater samples during the baseline and follow-up studies. There was more resistance to five antibiotics (tetracycline, ciprofloxacin, amoxicillin–clavulanic, aztreonam, and cefuroxime) but less resistance to gentamicin and meropenem in the follow-up study.

### 3.3. ESBL E. coli Loads in Wastewater Samples from the Legon STP, Accra, March–May 2023

ESBL *E. coli* were isolated in all influent and effluent samples of the follow-up study; 7.4% and 24% of *E. coli* isolated from influent and effluent samples, respectively, were ESBL positive.

## 4. Discussion

This before-and-after study was carried out following the recommendations to increase the efficiency of the Legon STP, which aimed at reducing *E. coli* concentrations in effluent wastewater. It was the first study to monitor changes in bacterial counts and their resistance patterns in an STP in Ghana following sewage treatment. Despite several efforts to disseminate the findings of the baseline study, only the increase in sewage intake (volume) materialised, though it was independent of the baseline study recommendation. Following this strategy, the follow-up study showed a large reduction in *E. coli* load in the effluent samples, although this was not statistically significant (*p* = 0.42) from the baseline study levels (99% efficiency for both). Despite this change, effluent *E. coli* counts still did not meet Ghana standards for effluent release into the environment. In addition, the follow-up study showed that resistance to certain antibiotics and the ESBL *E. coli* concentration in the effluent increased after passing through the treatment plant. Specifically, tetracycline, ciprofloxacin, cefuroxime, and amoxicillin–clavulanic showed increased percentages of resistance in the effluent compared to the influent.

These findings are important as they confirm that the bacterial removal efficiency of a properly functioning STP can reduce contamination in the environment, similar to other studies [14,20]. It also confirms previous findings that water processed through an STP can lead to the development of increased resistance to certain antibiotics [21]. Effluent *E. coli*, especially those with increased antibiotic resistance, pose a public health risk since the Onyasia Stream, which receives effluent from the Legon STP, serves as a source of irrigation for farms along its banks, thereby increasing the potential for “farm-to-fork transmission” of antimicrobial resistant bacteria [22,23].

We also noted that the concentration of *E. coli* in the influent of the follow-up study was considerably lower than in the baseline study, although this was not a statistically significant finding. The follow-up study revealed increased levels of AMR in commonly used antibiotic classes among *E. coli* isolates from effluent as compared to influent wastewater samples. This contrasted with the baseline study [14], where antimicrobial resistance levels in effluent were lower than in influent wastewater. Similar to other reports [24], higher proportions of ESBL *E. coli* were detected in effluent waters compared to influent waters following processing in the STP (follow-up study). These findings are consistent with reports that have described STPs as potential “hotspots” [7,25] for the proliferation and horizontal transfer of antimicrobial-resistant genes among microorganisms [8]. Other studies have shown that there is increased AMR in treatment plants where human and animal [8] waste, as well as waste from other sources, are processed together [25,26].

This study has several strengths. One is that the two studies took place in the same STP, which used the same treatment processes on both occasions. Another is that the water sampling and culture processing procedures were identical in both studies. Data management was consistent, and data were double-entered into Excel spreadsheets. Finally, the conduct and reporting of the study were in accordance with the STROBE (Strengthening the Reporting of Observational Studies in Epidemiology) guidelines [27,28].

However, we recognise some limitations. Studying only one plant meant that the findings might not be applicable to other plants. There was no accommodation for seasonal variation in influent water supplied to the STP. With a descriptive methodology and other possible variables contributing to the outcomes, we cannot attribute cause and effect to the contribution of the increased sewage influent.

There are several policy implications from this study. First, it appears that increasing the capacity (volume) of an STP maintains good clearance of *E. coli*. This reinforces investing in upgrading the other STPs in Accra and increasing connection to wastewater sewage systems to achieve maximum efficiency for the plants. Second, it is worrisome that passage through an STP increases the concentration of resistant *E. coli*, especially ESBL; there may be implications for the downstream environment. This suggests that there may be a need to employ other treatment modalities such as ozonation, filtration, and chemicals in addition to the current techniques [29,30,31] to render the effluent safe for environmental discharge. Third, it is important to continue to monitor STP effluents for changes in antibiotic resistance trends and to link them with AMR found in humans.

## 5. Conclusions

This before-and-after study confirmed the value of increasing the efficiency (increasing influent) of an STP in Accra, Ghana, and points the way to reducing bacterial discharge into the environment through investment in STPs and maximising their efficiency. It also suggests that ongoing surveillance of the antibiotic resistance patterns of *E. coli* is warranted, given the increase in resistance patterns following sewage processing.

## Figures and Tables

**Table 1:** *E. coli* load (median, IQR) in influent and effluent wastewater samples from the Legon sewage treatment plant, Accra, during the baseline (January 2018–June 2018) and follow-up (March to May 2023) studies.

Source	January–June 2018	March–May 2023	*p*-Value *
	*E. coli*	*E. coli*	
	Median (IQR) CFU/100 mL	Median (IQR) CFU/100 mL	
Influent	85 × 10^6^ (70–100 × 10^6^)	6.09 × 10^6^ (4–16 × 10^6^)	0.010 *
Effluent	380 (100–1300)	198.50 (106–342)	0.42 *
Plant Efficiency = $\frac{\left(Influent\right)-\left(Effluent\right)}{\left(Influent\right)}$ × 100	99%	99%	

CFU—Colony forming unit. * Wilcoxon rank Test. IQR—Interquartile range. The Ghana standard for *E. coli* after sewage treatment is 10.

## Data Availability

Requests to access these data should be sent to the corresponding author.
